# Dopaminergic Pathway Genes Influence Adverse Events Related to Dopaminergic Treatment in Parkinson's Disease

**DOI:** 10.3389/fphar.2019.00008

**Published:** 2019-01-28

**Authors:** Sara Redenšek, Dušan Flisar, Maja Kojović, Milica Gregorič Kramberger, Dejan Georgiev, Zvezdan Pirtošek, Maja Trošt, Vita Dolžan

**Affiliations:** ^1^Pharmacogenetics Laboratory, Institute of Biochemistry, Faculty of Medicine, University of Ljubljana, Ljubljana, Slovenia; ^2^Department of Neurology, University Medical Centre Ljubljana, Ljubljana, Slovenia

**Keywords:** Parkinson's disease, genetic polymorphism, dopaminergic pathway, personalized medicine, adverse events

## Abstract

Dopaminergic pathway is the most disrupted pathway in the pathogenesis of Parkinson's disease. Several studies reported associations of dopaminergic genes with the occurrence of adverse events of dopaminergic treatment. However, none of these studies adopted a pathway based approach. The aim of this study was to comprehensively evaluate the influence of selected single nucleotide polymorphisms of key dopaminergic pathway genes on the occurrence of motor and non-motor adverse events of dopaminergic treatment in Parkinson's disease. In total, 231 Parkinson's disease patients were enrolled. Demographic and clinical data were collected. Genotyping was performed for 16 single nucleotide polymorphisms from key dopaminergic pathway genes. Logistic and Cox regression analyses were used for evaluation. Results were adjusted for significant clinical data. We observed that carriers of at least one *COMT* rs165815 C allele had lower odds for developing visual hallucinations (OR = 0.34; 95% CI = 0.16–0.72; *p* = 0.004), while carriers of at least one *DRD3* rs6280 C allele and CC homozygotes had higher odds for this adverse event (OR = 1.88; 95% CI = 1.00–3.54; *p* = 0.049 and OR = 3.31; 95% CI = 1.37–8.03; *p* = 0.008, respectively). Carriers of at least one *DDC* rs921451 C allele and CT heterozygotes had higher odds for orthostatic hypotension (OR = 1.86; 95% CI = 1.07–3.23; *p* = 0.028 and OR = 2.30; 95% CI = 1.26–4.20; *p* = 0.007, respectively). Heterozygotes for *DDC* rs3837091 and *SLC22A1* rs628031 AA carriers also had higher odds for orthostatic hypotension (OR = 1.94; 95% CI = 1.07–3.51; *p* = 0.028 and OR = 2.57; 95% CI = 1.11–5.95; *p* = 0.028, respectively). Carriers of the *SLC22A1* rs628031 AA genotype had higher odds for peripheral edema and impulse control disorders (OR = 4.00; 95% CI = 1.62–9.88; *p* = 0.003 and OR = 3.16; 95% CI = 1.03–9.72; *p* = 0.045, respectively). Finally, heterozygotes for *SLC22A1* rs628031 and carriers of at least one *SLC22A1* rs628031 A allele had lower odds for dyskinesia (OR = 0.48; 95% CI = 0.24–0.98, *p* = 0.043 and OR = 0.48; 95% CI = 0.25–0.92; *p* = 0.027, respectively). Gene-gene interactions, more specifically *DDC-COMT, SLC18A2-SV2C*, and *SLC18A2-SLC6A3*, also significantly influenced the occurrence of some adverse events. Additionally, haplotypes of *COMT* and *SLC6A3* were associated with the occurrence of visual hallucinations (AT vs. GC: OR = 0.34; 95% CI = 0.16–0.72; *p* = 0.005) and orthostatic hypotension (ATG vs. ACG: OR = 2.48; 95% CI: 1.01–6.07; *p* = 0.047), respectively. Pathway based approach allowed us to identify new potential candidates for predictive biomarkers of adverse events of dopaminergic treatment in Parkinson's disease, which could contribute to treatment personalization.

## Introduction

Dopamine deficiency resulting from dopaminergic neuron death in the nigrostriatal pathway is the primary chemical disease hallmark of Parkinson's disease (PD). This neurodegenerative disorder presents with motor and non-motor symptoms (Kalia and Lang, 2015; Poewe et al., 2017). Treatment is symptomatic and is based on different dopamine replacement strategies. The most commonly used drugs in PD management are the dopamine receptor agonists (DAs) and the dopamine precursor levodopa (Connolly and Lang, 2014). The treatment of motor symptoms is effective, but adverse events (AEs) are rather common. The most frequent AEs caused by pulsatile levodopa treatment are motor fluctuations and dyskinesia. They are primarily related to levodopa dose and disease severity and usually occur after long-term levodopa treatment, but earlier occurrences may appear. Non-motor AEs, such as excessive daytime sleepiness (EDS) and sleep attacks, visual hallucinations (VHs), nausea/vomiting, orthostatic hypotension (OH), peripheral edema (PE), and impulse control disorders (ICDs) occur less often, usually shortly after treatment initiation and are associated with DAs and less commonly with levodopa (Chou, 2008; Antonini et al., 2009; Wood, 2010; Connolly and Lang, 2014; You et al., 2018). The occurrence of AEs in an individual currently can not be predicted.

Dopaminergic pathway is the most disrupted neurotransmitter pathway in the pathogenesis of PD. Moreover, polymorphic genes encode several enzymes, transporters, and receptors leading to inter-individual variability in the capacity of dopamine synthesis, transport, degradation, and signaling. Dopamine is synthesized in the dopaminergic neurons from tyrosine via levodopa to dopamine by dopa decarboxylase (*DDC*). It is then transported into synaptic vesicles through vesicular monoamine transporter 2 (*SLC18A2*). Dopamine is released to the synaptic cleft by exocytosis, where the synaptic vesicle glycoprotein 2C (*SV2C*) is involved. The signal is then transduced by dopamine receptors (*DRD1-5*) on postsynaptic neurons or glial cells. Reuptake of dopamine is facilitated via dopamine transporter (*SLC6A3*) back to the presynaptic neuron, where it gets repackaged into vesicles for future release or metabolized by monoamine oxidase B (*MAOB*) and catechol-O-methyltransferase (*COMT*) (Rang et al., 2012; Juarez Olguin et al., 2016; Nishijima and Tomiyama, 2016; You et al., 2018).

Dopamine precursor levodopa as the gold standard treatment of PD is transported through the blood brain barrier by the large neutral amino acid transporter (*SLC7A5*) and is converted to dopamine in dopaminergic neurons. It can get metabolized also in peripheral tissues by DDC and COMT (Nishijima and Tomiyama, 2016; You et al., 2018). Transport, distribution, and elimination of the drug is facilitated by the transporter SLC22A1 (Becker et al., 2011).

Several pharmacogenomics studies searching for associations between single nucleotide polymorphisms (SNPs) of above mentioned genes and response to dopaminergic treatment have already been performed and some significant results have been reported. *DRD1-4* SNPs have already been associated with the occurrence of several AEs of dopaminergic treatment, such as dyskinesia (Zappia et al., 2005; Strong et al., 2006; Lee et al., 2011; Rieck et al., 2012), ICDs (Lee et al., 2009; Zainal Abidin et al., 2015; Krishnamoorthy et al., 2016), nausea/vomiting (Rieck et al., 2016), sleep attacks (Paus et al., 2004; Rissling et al., 2004), and VHs (Goetz et al., 2001). Furthermore, variability of transporter genes has already been associated with drug response. *SLC6A3* SNPs have shown associations with dyskinesia (Kaiser et al., 2003; Kaplan et al., 2014), VHs (Schumacher-Schuh et al., 2013), and motor response to acute levodopa challenge (Moreau et al., 2015). SNPs of *SLC22A1* and *SV2C* influenced the levodopa dose (Becker et al., 2011; Altmann et al., 2016). The most studied *COMT* SNP rs4680 has already been associated with motor fluctuations (Watanabe et al., 2003; Hao et al., 2014; Wu et al., 2014), dyskinesia (Watanabe et al., 2003; Bialecka et al., 2008; de Lau et al., 2012), and daytime sleepiness (Frauscher et al., 2004). Furthermore, *MAOB* rs1799836 has also been associated with the occurrence of dyskinesia (Hao et al., 2014), while *DDC* rs921451 and rs3837091 have been associated with the motor response to acute levodopa challenge (Devos et al., 2014). The major limitation of all these studies was that they included only individual genes involved in dopaminergic pathway.

The aim of this study was to comprehensively evaluate the influence of selected SNPs of key dopaminergic pathway genes on the occurrence of motor and non-motor AEs of dopaminergic treatment in PD.

## Materials and Methods

### Study Participants

A total of 231 unrelated PD patients were enrolled in this retrospective cohort study. Patients were recruited as they were coming for their regular appointment at the Department of Neurology, University Medical Centre Ljubljana, Slovenia between October 2016 and April 2018. Inclusion criteria were (1) diagnosis of PD according to the UK Parkinson Disease Society Brain Bank criteria (Goetz et al., 2008) by an experienced movement disorders specialist, (2) available clinical data, (3) at least 3 months of levodopa and/or DAs treatment duration, (4) ongoing dopaminergic therapy with levodopa and/or DAs. Patients with atypical and secondary forms of parkinsonisms were not included in the study.

Patients and their caregivers underwent a structured interview to obtain demographic and clinical data. Additional information was obtained from the medical records. We focused on eight main AEs of dopaminergic treatment as primary endpoints: motor fluctuations, dyskinesia, EDS and sleep attacks, VHs, nausea/vomiting, OH, PE, and ICDs. The AE was defined as absent or present according to clinical examination, clinical documentation, and patients' answers to specific questions.

The study protocol was approved by the Slovenian Ethics Committee for Research in Medicine (KME 42/05/16). All subjects gave written informed consent in accordance with the Declaration of Helsinki.

### SNP Selection

Ten candidate genes were included due to their putative involvement in the dopaminergic pathway (see Supplementary Figure 1) (Redenšek et al., 2018). Several methods were used for SNP selection. First, we searched the literature for SNPs in dopaminergic genes that were already found to be associated with dopaminergic treatment response and the occurrence of AEs. We have also searched for genes involved in dopaminergic pathway (Kanehisa and Goto, 2000) (dopamine synthesis, transport, degradation, and signaling) and their functional SNPs residing in the promoter or coding regions (Sherry et al., 2001). Additionally, we used the SNP function prediction tool (Xu and Taylor, 2009) to select SNPs for the analysis based on their predicted function.

### DNA Isolation and Genotyping

Peripheral blood samples were obtained for DNA extraction. Genomic DNA was isolated using the FlexiGene DNA Kit (Qiagen, Hilden, Germany) according to the manufacturer's protocol. Genotyping was performed for 16 SNPs. All of the studied SNPs were genotyped with KASPar assays (KBiosciences, Herts, UK and LGC Genomics, UK) according to manufacturer's instructions. Ten percent of samples were genotyped in duplicate as quality control and all the results were concordant.

### Statistical Analysis

Median and 25th to 75th percentile range were used to describe central tendency and variability of continuous variables, while frequencies were used to describe the distribution of categorical variables. The agreement of genotype frequencies with Hardy-Weinberg equilibrium was examined by chi-squared test. Logistic regression was used to calculate odds ratios (ORs) and 95% confidence intervals (95%CIs) to examine the associations of selected SNPs and clinical data with the risk for AEs. Dominant, additive, and recessive genetic models were used for analysis depending on the genotype frequencies. Males and females were analyzed separately for *MAOB* rs1799836 due to its X chromosome location.

The influence of SNPs on the time to occurrence of motor AEs after levodopa treatment initiation was evaluated by the Cox proportional hazards model to calculate hazard ratio (HR) and the 95%CI.

Gene-gene interactions were examined with logistic regression analysis. The model included two polymorphisms and their interactive term to calculate the OR, 95%CI and *p*-value for each gene–gene interaction.

A haplotype analysis was carried out to assess the combined effect of multiple SNPs in the same gene. On the basis of genotype data, haplotypes were reconstructed and analyzed using the Thesias program (Tregouet and Garelle, 2007). Only haplotypes with frequencies above 5% were included in the analysis. The most frequent haplotype was used as reference.

All statistical tests were two-sided. Bonferroni correction was used to account for multiple comparisons to prevent false positive results. For genetic data *p*-values up to 0.0036 (0.05/14) were considered statistically significant, while p values between 0.0036 and 0.0500 were considered nominally significant. For clinical data *p*-values up to 0.0056 (0.05/9) were considered statistically significant, while *p*-values between 0.0056 and 0.0500 were considered nominally significant. For an allelic variant with minor allele frequency 0.34 and with a 32% prevalence of an AE, this study had 80% or more power to detect OR of 0.36 or less and OR of 2.31 or more. Power calculation was conducted by the PS Power and sample size calculations, version 3.0. All statistical analyses were carried out by IBM SPSS Statistics, version 21.0 (IBM Corporation, Armonk, NY, USA).

## Results

### Patients' Characteristics

Patients' median age at enrolment was 72.5 years (65.7–78.0) and median dopaminergic treatment duration was 7.3 years (3.6–13.5). In total, 200 (86.6%) patients experienced at least one of the AEs. The proportion of patients reporting an individual AE varied from 13.9 to 53.2%. On average, 32.2% of patients experienced a specific AE. Demographic and clinical characteristics of patients are presented in Table 1 along with the list and frequencies of AEs.

**Table 1:** Demographic and clinical data of PD patients with the list of adverse events.

**Characteristic**		**All patients (*N* = 231)**
Gender	Male (%)	132 (57.1)
	Female (%)	99 (42.9)
Side of disease initiation	Left (%)	91 (39.4)
	Both (%)	21 (9.1)
	Right (%)	119 (51.5)
Tremor-predominant PD	No (%)	46 (20.0)
	Yes (%)	185 (80.0)
Ever being treated with DAs^b^	No (%)	57 (25.1)
	Yes (%)	170 (74.9)
Age at diagnosis	Median (25%–75%), years	62.2 (54.8–71.7)
Disease duration	Median (25%–75%), years	7.6 (3.8–13.6)
Dopaminergic treatment duration^e^	Median (25%−75%), years	7.3 (3.6–13.5)
Levodopa treatment duration^d^	Median (25%–75%), years	6.1 (2.3–11.1)
LED at enrolment^a^, ^c^	Median (25%–75%), mg/day	975 (600–1363.5)
**Adverse event**	**Number (%) of patients experiencing the adverse event**	
Motor fluctuations	123 (53.2)	
Dyskinesia	101 (43.7)	
EDS and sleep attacks	81 (35.1)	
Visual hallucinations^f^	57 (24.7)	
Nausea/vomiting^f^	70 (30.3)	
Orthostatic hypotension^f^	87 (37.7)	
Peripheral edema^f^	44 (19.0)	
Impulse control disorders^f^	32 (13.9)	

a *LED calculated according to Tomlinson et al. (2010)*.

b *Data missing for four patients*.

c *Data missing for five patients*.

d *Data missing for seven patients*.

e *Data missing for three patients*.

f *Data missing for one patient*.

### SNP Genotyping Analysis

Sixteen SNPs were selected for the analysis. *COMT* rs4680 (p.Val158Met), *DRD2* rs1799732 (c.-486_-485insC), and *DRD3* rs6280 (p.Gly9Ser) have previously already been associated with the occurrence of AEs in PD treatment according to the literature. Additionally, one functional nonsynonymous SNP rs165815 (p.Arg900Gln) residing in the coding region was selected in the *COMT* gene. Furthermore, two functional SNPs were selected in genes *DRD2* (rs1801028 - p.Ser311Cys) (He et al., 2016), and *SLC22A1* (rs628031 - p.Met216Val). Two SNPs in *DDC* (rs921451 - c.-29+5426A>G and rs3837091 - c.-61_-58delAGAG), one in *MAOB* (rs1799836 - c.1348-36A>G), and one in *SLC6A3* (rs393795 - c.653+4065C>A) were selected due to their previous association with drug response in PD. Additionally, two *SLC6A3* SNPs (rs6347 - p.Ser405 = and rs104209 - c.^*^35T>C), two *SLC7A5* SNPs (rs1060253 - c.^*^438C>G and rs1060257 - c.^*^1282G>A), one *SLC18A2* SNP (rs14240 - c.^*^294T>A), and one *SV2C* SNP (rs1423099 - c.-58C>T) were selected with the SNP function prediction tool (Xu and Taylor, 2009).

Investigated SNPs and genotype distributions are presented in Supplementary Table 1. Genotype frequencies did not deviate from Hardy-Weinberg equilibrium (HWE) in the majority of cases (*p* > 0.05). In the case of *DDC* rs921451 and rs3837091 the frequencies did not match the HWE requirements. However, frequencies for these two SNPs were not significantly different than frequencies reported in the HapMap-CEU population (*p* = 0.657 and *p* = 0.120, respectively). On the other hand, frequencies of *SLC7A5* rs1060253 and rs1060257 deviated from HWE and from the HapMap-CEU genotype distributions significantly, so we excluded them from further analysis. None of the SNPs included in the final analysis are in linkage disequilibrium.

### Influence of Genetic Variability on the Risk for Adverse Events

Univariate logistic regression identified some possible associations between SNPs and AEs. The significant and nominally significant associations are presented in Table 2, but all associations are given in the Supplementary Tables 2–4. We observed that carriers of at least one *COMT* rs165815 C allele had lower odds for developing VHs (*p* = 0.006). Carriers of at least one *DRD3* rs6280 C allele had almost two times higher odds (*p* = 0.033), whereas CC homozygotes had more than three times higher odds for developing VHs (*p* = 0.006). Genotypes of the *DDC* SNPs showed associations with the occurrence of OH. Carriers of at least one *DDC* rs921451 C allele had almost two times higher odds for developing this AE (*p* = 0.033), whereas under additive model only heterozygotes presented with a nominally significant association (*p* = 0.009). *DDC* rs3837091 heterozygotes also had higher odds for OH (*p* = 0.048). *SLC22A1* rs628031 AA carriers had higher odds for developing OH (*p* = 0.034), peripheral edema (*p* = 0.003), and ICDs (*p* = 0.028). On the other hand, *SLC22A1* rs628031 heterozygotes had lower odds for presenting with motor fluctuations (*p* = 0.007) and dyskinesia (*p* = 0.003). Furthermore, under dominant model carriers of at least one *SLC22A1* rs628031 A allele had lower odds for the occurrence of dyskinesia (*p* = 0.007). Dyskinesia was also more likely to develop in *DDC* rs3837091 heterozygotes (*p* = 0.037). Two of the above listed associations with the *p* = 0.003 were treated as statistically significant results, whereas other associations were treated as nominally significant.

**Table 2:** Significant and nominally significant associations of the univariate and multivariate logistic analyses of genetic factors with adverse events.

**Gene**	**Genotype**	**OR**	**95% CI**	***p*-value**	**Adjusted for**	OR adj.^*^	**95% CI**	***p*-value**
**VISUAL HALLUCINATIONS**								
*COMT* rs165815	TT	Ref.				Age at diagnosis	Ref.	
	CC + CT^*^	**0.36**	**0.18–0.74**	**0.006**		**0.34**	**0.16-0.72**	**0.004**
*DRD3* rs6280	TT	Ref.					Ref.	
	TC	1.59	0.81–3.12	0.174		1.53	0.77-3.02	0.226
	CC	**3.40**	**1.43–8.12**	**0.006**		**3.31**	**1.37-8.03**	**0.008**
	TC + CC	**1.96**	**1.05–3.65**	**0.033**		**1.88**	**1.00-3.54**	**0.049**
**ORTHOSTATIC HYPOTENSION**								
*DDC* rs921451	TT	Ref.				Age at diagnosis	Ref.	
	CT	**2.23**	**1.23–4.06**	**0.009**		**2.30**	**1.26-4.20**	**0.007**
	CC	1.17	0.54–2.52	0.695		1.17	0.54-2.53	0.693
	CT+CC	**1.82**	**1.05–3.16**	**0.033**		**1.86**	**1.07-3.23**	**0.028**
*DDC* rs3837091	AGAGAGAG	Ref.					Ref.	
	AGAG-	**1.79**	**1.00–3.19**	**0.048**		**1.94**	**1.07-3.51**	**0.028**
	–	0.96	0.40–2.31	0.926		0.93	0.39-2.26	0.880
	AGAG- + –	1.54	0.90–2.62	0.117		1.60	0.93-2.76	0.089
*SLC22A1* rs628031	GG	Ref.					Ref.	
	GA	1.03	0.57–1.84	0.929		0.98	0.54-1.76	0.946
	AA	**2.46**	**1.07-5.66**	**0.034**		**2.57**	**1.11-5.95**	**0.028**
	GA + AA	1.26	0.73-2.17	0.399		1.01	0.99-1.03	0.375
**PERIPHERAL EDEMA**								
*SLC22A1* rs628031	GG	Ref.				Age at diagnosis	Ref.	
	GA	0.88	0.41-1.90	0.752		0.86	0.40-1.87	0.708
	AA	**3.92**	**1.60-9.63**	**0.003**		**4.00**	**1.62-9.88**	**0.003**
	GA + AA	1.38	0.70-2.72	0.353		1.38	0.70-2.73	0.352
**IMPULSE CONTROL DISORDERS**								
*SLC22A1* rs628031	GG	Ref.				Ever being treated with DAs Age at diagnosis	Ref.	
	GA	1.40	0.59–3.32	0.445		1.97	0.77–5.01	0.156
	AA	**3.20**	**1.13–9.06**	**0.028**		3.16	1.03–9.72	**0.045**
	GA + AA	1.76	0.79–3.91	0.165		2.27	0.96–5.41	0.064
**MOTOR FLUCTUATIONS**								
*SLC22A1* rs628031	GG	Ref.				Side of disease initiation Tremor-predominant PD Ever being treated with DAs Age at diagnosis	Ref.	
	GA	**0.46**	**0.26-0.81**	**0.007**		0.56	0.28-1.11	0.095
	AA	1.62	0.67-3.88	0.282		1.50	0.53-4.23	0.446
	GA + AA	0.61	0.36-1.04	0.070		0.70	0.37-1.34	0.279
**DYSKINESIA**								
*DDC* rs3837091	AGAGAGAG	Ref.				Tremor-predominant PD Ever being treated with DAs Age at diagnosis	Ref.	
	AGAG-	**1.83**	**1.04-3.24**	**0.037**		1.21	0.60-2.45	0.590
	–	0.73	0.31-1.75	0.480		0.65	0.26-1.75	0.427
	AGAG- + –	1.46	0.87-2.46	0.156		1.04	0.54-1.99	0.905
*SLC22A1* rs628031	GG	Ref.					Ref.	
	GA	**0.43**	**0.24-0.75**	**0.003**		**0.48**	**0.24-0.98**	**0.043**
	AA	0.70	0.31-1.57	0.387		0.46	0.17-1.25	0.126
	GA + AA	**0.48**	**0.28-0.82**	**0.007**		**0.48**	**0.25-0.92**	**0.027**

* *Recessive model was used*.

Significant and nominally significant associations are written in bold text.

As shown in the Table 3, several clinical parameters were included in our analysis: gender, side of disease initiation, tremor-predominant PD, ever being treated with DAs, age at diagnosis, disease duration, dopaminergic treatment duration, levodopa treatment duration, and LED at enrolment (calculated according to Tomlinson et al., 2010). After univariate logistic regression of these parameters we identified clinical parameters that significantly or nominally significantly affect the occurrence of each AE. These clinical parameters were then used for adjustment of significant or nominally significant genetic factors in multivariate analysis. When considering continuous clinical data only age at diagnosis was used for adjustment due to collinearity of all five continuous parameters. Most of the associations observed in univariate analysis retained their significance level after adjustment as shown in the Table 2.

**Table 3:** Univariate analysis of the influence of clinical parameters on the occurrence of adverse events.

	**EDS and sleep attacks**		**Visual hallucinations**		**Nausea and vomiting**		**Orthostatic hypotension**	
	**OR (95% CI)**	***p*-value**	**OR (95% CI)**	***p*-value**	**OR (95% CI)**	***p*-value**	**OR (95%CI)**	***p*-value**
Gender (male = ref.)	0.69 (0.40–1.320	0.190	0.73 (0.39–1.35)	0.311	**2.79 (1.56–4.97)**	**0.001**	0.73 (0.43–1.26)	0.264
Side of disease initiation (left = ref.)	0.74 (0.26–2.09)	0.566	0.35 (0.07–1.61)	0.176	0.89 (0.31–2.52)	0.820	1.57 (0.60–4.09)	0.356
	1.04 (0.59–1.85)	0.884	1.31 (0.70–2.47)	0.395	0.96 (0.53–1.74)	0.894	1.01 (0.57–1.79)	0.964
Tremor-predominant PD (No = ref.)	0.90 (0.46–1.77)	0.764	1.06 (0.50–2.26)	0.879	1.00 (0.50–2.02)	1.000	0.67 (0.35–1.28)	0.223
Ever being treated with DAs (No = ref.)	1.90 (0.97–3.75)	0.063	1.80 (0.84–3.84)	0.131	**2.99 (1.37–6.50)**	**0.006**	0.81 (0.44–1.49)	0.498
Age at diagnosis	1.00 (0.98–1.02)	0.744	**0.94 (0.94–0.99)**	**0.008**	**0.97 (0.95–1.00)**	**0.017**	1.01 (0.99–1.03)	0.343
Disease duration	**1.04 (1.00–1.08)**	**0.039**	**1.14 (1.09–1.21)**	** < 0.001**	1.02 (0.98-1.06)	0.405	**1.04 (1.00–1.08)**	**0.046**
Dopaminergic treatment duration	1.04 (1.00–1.08)	0.073	**1.13 (1.08–1.19)**	** < 0.001**	1.01 (0.97–1.06)	0.537	**1.05 (1.01–1.10)**	**0.015**
Levodopa treatment duration	1.04 (1.00–1.09)	0.071	**1.14 (1.08–1.20)**	** < 0.001**	1.00 (0.96–1.05)	0.997	**1.07 (1.03–1.12)**	**0.002**
LED at enrolment	**1.00 (1.00–1.00)**	**0.045**	**1.00 (1.00–1.00)**	**0.006**	1.00 (1.00–1.00)	0.670	**1.00 (1.00–1.00)**	**0.045**
	**Peripheral edema**		**Impulse control disorders**		**Motor fluctuations**		**Dyskinesia**	
	**OR (95% CI)**	***p*****-value**	**OR (95% CI)**	***p*****-value**	**OR (95% CI)**	***p*****-value**	**OR (95% CI)**	***p*****-value**
Gender (male = ref.)	0.64 (0.32–1.28)	0.206	0.57 (0.26–1.26)	0.165	0.95 (0.56–1.60)	0.849	1.05 (0.62–1.78)	0.848
Side of disease initiation (left = ref.)	2.37 (0.78–7.22)	0.129	0.33 (0.04–2.65)	0.294	**0.34 (0.12–0.96)**	**0.042**	0.56 (0.20–1.57)	0.269
	1.58 (0.76–3.29)	0.225	1.24 (0.57–2.70)	0.596	1.14 (0.66–1.98)	0.634	1.28 (0.74–2.22)	0.376
Tremor-predominant PD (No = ref.)	1.16 (0.50–2.69)	0.738	0.88 (0.35–2.17)	0.775	**0.42 (0.21–0.85)**	**0.015**	**0.33 (0.17–0.65)**	**0.001**
Ever being treated with DAs (No = ref.)	2.45 (0.98–6.14)	0.057	**12.00 (1.60–90.13)**	**0.016**	**7.28 (3.52–15.09)**	** < 0.001**	**5.72 (2.64–12.40)**	** < 0.001**
Age at diagnosis	1.00 (0.97–1.03)	0.988	**0.93 (0.90–0.96)**	** < 0.001**	**0.89 (0.86–0.92)**	** < 0.001**	**0.88 (0.85–0.92)**	** < 0.001**
Disease duration	1.01 (0.96–1.06)	0.671	1.04 (0.99–1.01)	0.111	**1.45 (1.32–1.60)**	** < 0.001**	**1.34 (1.24–1.44)**	** < 0.001**
Dopaminergic treatment duration	1.01 (0.96–1.06)	0.626	1.04 (0.98–1.09)	0.180	**1.45 (1.32–1.59)**	** < 0.001**	**1.33 (1.23–1.43)**	** < 0.001**
Levodopa treatment duration	1.03 (0.98–1.08)	0.320	1.00 (0.95–1.07)	0.885	**1.43 (1.30–1.58)**	** < 0.001**	**1.32 (1.22–1.42)**	** < 0.001**
LED at enrolment	1.00 (1.00–1.00)	0.247	1.00 (1.00–1.00)	0.162	**1.01 (1.00–1.01)**	** < 0.001**	**1.00 (1.00–1.00)**	** < 0.001**

*Significant and nominally significant associations are written in bold text*.

### Influence of Genetic Variability on the Time to Occurrence of Motor Adverse Events

We were able to obtain data on the time to occurrence of motor AEs after levodopa treatment initiation. For genetic factors significantly associated with AEs after univariate logistic regression we performed the survival analysis. None of the investigated genetic factors showed any significant effect on the time to occurrence of motor AEs after levodopa treatment initiation. These results are presented in the Supplementary Figure 2.

### Influence of Gene-Gene Interactions on the Risk for Adverse Events

According to the structure of dopaminergic pathway, we selected gene pairs that could interact in affecting the occurrence of AEs according to their physiological function. We tested the following gene pairs: *COMT-DDC, SLC18A2–SV2C, DRD2–DRD3*, and *SLC6A3–SLC18A2*. Our results suggest that patients with at least one *DDC* rs921451 C allele and at least one *COMT* rs165815 C allele had lower odds for developing VHs (OR = 0.16, 95% CI = 0.03–0.78, *p* = 0.023). Furthermore, patients with at least one *DDC* rs3837091 deletion allele and at least one *COMT* rs165815 C allele had lower odds for developing EDS and sleep attacks (OR = 0.28, 95% CI = 0.09–0.94, *p* = 0.039). Nausea/vomiting were less likely to occur in patients with at least one *SLC18A2* rs14240 C allele and at least one *SV2C* rs1423099 T allele (OR = 0.18, 95%CI = 0.05–0.74, *p* = 0.017). Moreover, nausea/vomiting were also less likely to occur in patients with at least one *SLC18A2* rs14240 C allele and *SLC6A3* rs393795 T allele (OR = 0.15, 95% CI = 0.04–0.66, *p* = 0.012). The presented associations are nominally significant.

### Influence of Haplotypes on the Risk for Adverse Events

Only two genes' haplotypes showed nominally significant associations, namely *COMT* and *SLC6A3*. All four *COMT* haplotypes (AT, GT, GC, and AC) were included in the analysis. VHs were less likely to occur in patients with GC haplotype compared to the most common AT haplotype (*p* = 0.005). Five *SLC6A3* haplotypes (ATG, GCG, ATT, ACG, and GTG) were analyzed. They covered 95% of genetic variability. Patients with the ACG haplotype had higher odds for developing OH in comparison to patients with the ATG haplotype (*p* = 0.047). Three *DDC* haplotypes (TAGAG, C-, and CAGAG) were included in the analysis and they together covered 100% of the genetic variability, but none of them showed any significant association with any of the AEs. Finally, two *DRD2* haplotypes (CC, C-) covering 97% of genetic variability were analyzed, but no significant association was found. Nominally significant results are presented in Table 4, whereas complete results are presented in Supplementary Tables 5–8. No significant associations were observed.

**Table 4:** Significant associations between haplotypes and adverse events.

***COMT***	**Visual hallucinations**		***SLC6A3***	**Orthostatic hypotension**	
**Haplotype**	**OR (95%CI)**	***p*-value**	**Haplotype**	**OR (95%CI)**	***p*-value**
AT	Ref.			ATG	Ref.
GT	0.77 (0.47–1.23)	0.270	GCG	1.27 (0.73–2.21)	0.393
GC	**0.34 (0.16–0.72)**	**0.005**	ATT	1.31 (0.72–2.40)	0.383
AC	0.42 (0.11–1.65)	0.217	ACG	**2.48 (1.01–6.07)**	**0.047**
			GTG	0.80 (0.27–2.37)	0.690

*Nominally significant associations are written in bold text*.

## Discussion

In the present study, we examined the association of 14 selected SNPs from dopaminergic pathway genes with AEs related to dopaminergic treatment in PD. We would like to point out two most important findings. First, a very strong effect of genetic variability in the *COMT* gene on the occurrence of VHs was pointed out in the logistic regression, gene-gene interaction, and haplotype analyses. The *COMT* rs165815 C allele appeared to be protective against this AE. Another SNP showing strong association was the *SLC22A1* rs628031 as its genotype significantly or nominally significantly influenced the occurrence of five AEs: OH, PE, ICDs, motor fluctuations, and dyskinesia. Therefore, these two SNPs seem to be good candidate biomarkers for AEs of dopaminergic treatment in PD.

*COMT* rs165815 showed a nominally significant protective effect on the VHs' occurrence, whereas *COMT* rs4680 did not show any association with the studied AEs. The role of the *COMT* rs4680 has already been thoroughly examined in PD patients. In contrast with our non-significant findings, *COMT* rs4680 was associated with the occurrence of dyskinesia, motor fluctuations (Watanabe et al., 2003; Bialecka et al., 2008; de Lau et al., 2012; Hao et al., 2014; Wu et al., 2014), and daytime sleepiness (Frauscher et al., 2004) in other studies. The *COMT* rs165815 has never been studied in PD, but has already been studied in connection to treatment-resistant schizophrenia (Terzić et al., 2016).

Genetic variability of *DDC* was also associated with the development of AEs. Nominally significant associations were observed between *DDC* genotypes and OH development in a way that certain genotypes increased risk for the AE's occurrence. Devos et al. (2014) suggested that the studied two SNPs reduce DDC expression or activity. Low enzyme activity leads to low norepinephrine concentration, which may result in vasodilatation and consequently hypotension (Lau et al., 2018). This route of hypotension development is supported by basic agonistic effect of dopaminergic drugs on dopamine receptors, which also leads to vasodilatation. Furthermore, *DDC* rs3837091 heterozygotes were also more likely to develop dyskinesia. It is plausible that reduced DDC activity may result in higher levodopa bioavailability in the central nervous system and could thus cause dyskinesia. The association did not retain the significance level after adjustment, though. Both *DDC* SNPs were already associated with motor response to acute levodopa treatment in a previous study (Devos et al., 2014), but have never been associated with AEs of dopaminergic treatment.

Several AEs were associated with genetic variability in the *SLC22A1*. The rs628031 AA genotype increased risk for OH, PE, and ICDs, whereas GA genotype appeared to be protective against dyskinesia. The association between this SNP and motor fluctuations did not remain significant after adjustment. *SLC22A1* rs622342 has been associated with levodopa dose previously (Becker et al., 2011), but *SLC22A1* rs628031 has never been examined before in association with PD. Since this SNP appeared to be important in the occurrence of several AEs, it may play a role in the overall drug action and may modify the overall AEs' susceptibility.

*DRD3* rs6280 CC genotype was observed as a risk factor for VHs. Similar finding was already reported in the study by Goetz et al. (2001). This SNP may lead to modifications in intracellular signaling via higher binding affinity (Krishnamoorthy et al., 2016; Rieck et al., 2016). We did not confirm the results of some previously published data, such as the association between this SNP and ICDs (Lee et al., 2009; Krishnamoorthy et al., 2016), dyskinesia (Lee et al., 2011), and nausea/vomiting (Rieck et al., 2016), however all of the studied populations differed from our patient cohort in ethnicity (Indian, Korean, and Brazilian, respectively).

We were also not able to confirm the results of some previous studies in other populations. We did not confirm the association between *DRD2* rs1799732 and dyskinesia (Rieck et al., 2012) and *DRD2* rs1799732 and nausea/vomiting (Rieck et al., 2016) reported in Brazilian patients. We also did not find an association between *SLC6A3* rs393795 and dyskinesia as reported in Israeli patients (Kaplan et al., 2014) and the association between *MAOB* rs1799836 and dyskinesia reported in Chinese patients (Hao et al., 2014).

We also examined the possible influence of significant genetic parameters on the time to occurrence of motor complications after levodopa treatment initiation. No significant associations were found. It is possible that we may have missed this association because the inclusion criterion regarding the treatment duration was set to 3 months, which is rather short. Therefore, some patients could develop AEs later. However, we found some patients experiencing motor complications from the treatment initiation onwards.

We observed a significant influence of the gene-gene interaction *COMT*-*DDC* on the occurrence of VHs. Association of *COMT* rs165815 with VHs was already detected by univariate regression analysis. According to some publications *DDC* rs921451 may decrease the enzyme's function (Devos et al., 2014), which could lead to reduced dopamine concentrations in the central nervous system. As VHs presumably develop due to elevated dopaminergic stimulation (Rolland et al., 2014), the interaction between *DDC* and *COMT* SNPs indeed could lead to reduced odds for this AE. Also EDS and sleep attacks could arise due to elevated dopaminergic stimulation (Knie et al., 2011), which supports the effect of *DDC* rs3837091 and *COMT* rs165815 interaction on this AE occurrence as well. The interaction between *SLC18A2* rs14240 and *SV2C* rs1423099 significantly affects the occurrence of nausea/vomiting. Furthermore, the interaction between *SLC18A2* rs14240 and *SLC6A3* rs393795 significantly influences the occurrence of the same AE. Functional background must still be determined, but might be related to area postrema originating nausea/vomiting (Morris, 1978).

The haplotype analysis additionally supported the observation of *COMT* genetic variability being involved in VHs' occurrence. According to our results, *COMT* rs165815 might be a good candidate for a genetic biomarker of protection against this AE. *SLC6A3* did not show any associations with AEs in the above described analyses, but a more comprehensive haplotype analysis revealed that the ACG haplotype carriers have more than two times higher odds for developing OH compared to ATG haplotype carriers. This is the first finding of OH being related to this gene. More functional studies are warranted.

Although our study presents some novel findings, some limitations have to be considered. The sample is of moderate size, although it is comparable to the sample sizes of similar PD pharmacogenetic studies and the study power was calculated. Another limitation was that all of the AEs were analyzed as categorical variables. The use of clinical scales to evaluate the severity of various AEs would allow a more in depth analysis of possible associations. A prospective study would have a greater chance to detect even subtler relations between treatment and AEs. It should also be noted that our results should be validated in an independent sample, before they could be applied in a clinical setting.

However, several advantages of this study should be pointed out. All of the patients were recruited from one department, which means that patients were treated according to the same guidelines. Furthermore, these guidelines did not change during the recruitment period. Our study was not biased by genetic heterogeneity since all the patients were recruited in a geographic area with an ethnically homogeneous population (Vidan-Jeras et al., 1998; Mizzi et al., 2016). Study was designed according to a pathway approach, enabling a comprehensive analysis of genetic variability in the dopaminergic pathway.

## Conclusions

The results of this study further confirm the role of genetic variability in dopaminergic pathway in the development of AEs of dopaminergic treatment in PD and suggest some new possible predictive biomarkers of the studied AEs, such as *COMT* rs165815 and *SLC22A1* rs628031. Validation of these biomarkers in independent patient cohorts would enable prediction of AEs of dopaminergic treatment in PD and maybe someday also personalized treatment regimens to be implemented in PD management to minimize the burden that AEs present to PD patients.

## Author Contributions

All authors have made a substantial intellectual contribution to this work and approved its final version for submission. SR, MT, and VD formed the study focus and organized and executed the study. SR performed the experiments and statistical analysis under the supervision of MT and VD. SR wrote the first draft of the manuscript also under supervision of MT and VD. DF, MK, MG, DG, and ZP helped with patient enrolment and contributed to the final version of the manuscript.

This study was funded by the Slovenian Research Agency (ARRS), grant number P1-0170 and grant for young researchers (SR).

### Conflict of Interest Statement

The authors declare that the research was conducted in the absence of any commercial or financial relationships that could be construed as a potential conflict of interest.

## Abbreviations

PD: parkinson's disease
DAs: dopamine receptor agonists
AEs: adverse events
EDS: excessive daytime sleepiness
VHs: visual hallucinations
OH: orthostatic hypotension
ICDs: impulse control disorders
SNPs: single nucleotide polymorphisms
ORs: odds ratios
CIs: confidence intervals
HR: hazard ratio
HWE: hardy-weinberg equilibrium
PE: peripheral edema
LED: levodopa equivalent dose.
