# Supplementary material for: Dopaminergic Pathway Genes Influence Adverse Events Related to Dopaminergic Treatment in Parkinson's Disease
Source: Front Pharmacol. 2019 Jan 28;10:8. doi: 10.3389/fphar.2019.00008 (PMC6360186; doi:10.3389/fphar.2019.00008)
Supplement: Supplementary file 1 [file Data_Sheet_1.docx]

**Supplementary table 1: SNP characteristics and genotype distributions**

| **Gene** | **Polymorphism** | **MAF^f^** | **Location in gene** | **SNP function prediction** | **Genotype** | **N (%)** | **HWE**  **(p-value)** |
| --- | --- | --- | --- | --- | --- | --- | --- |
| *COMT* | rs4680  p.Val158Met | 0.37 | Coding region | Nonsynonymous may influence splicing**^b^** | GG | 61 (26.4) | 0.264 |
|  |  |  |  |  | GA | 107 (46.3) |  |
|  |  |  |  |  | AA | 63 (27.3) |  |
|  | rs165815  p.Arg900Gln | 0.38 | Coding region | Nonsynonymous may influence splicing**^b^** | CC | 9 (3.9) | 0,856 |
|  |  |  |  |  | CT | 71 (30.7) |  |
|  |  |  |  |  | TT | 151 (65.4) |  |
| *DDC* | rs921451  c.-29+5426A>G | 0.35 | Intron | Functional impact on expression^c^ | TT | 100 (43.3) | 0,007 |
|  |  |  |  |  | CT | 89 (38.5) |  |
|  |  |  |  |  | CC | 42 (18.2) |  |
|  | rs3837091  c.-61_-58delAGAG | 0.29 | Intron | Functional impact on expression^c^ | AGAGAGAG | 122 (52.8) | 0,015 |
|  |  |  |  |  | AGAG- | 81 (35.1) |  |
|  |  |  |  |  | -- | 28 (12.1) |  |
| *MAOB^a^* | rs1799836  c.1348-36A>G | 0.46 | Intron | Functional impact on expression and activity^d^ | A, AA | 106 (45.9) | 0.684^h^ |
|  |  |  |  |  | AG | 51 (22.5) |  |
|  |  |  |  |  | G, GG | 73 (31.6) |  |
| *SLC6A3* | rs6347  p.Ser405= | 0.30 | Coding region | May influence splicing and miRNA binding**^b^** | AA | 121 (52.4) | 0.311 |
|  |  |  |  |  | AG | 88 (38.1) |  |
|  |  |  |  |  | GG | 22 (9.5) |  |
|  | rs1042098  c.*35T>C | 0.30 | 3’UTR | May influence splicing**^b^** | TT | 124 (53.7) | 0.401 |
|  |  |  |  |  | TC | 87 (37.7) |  |
|  |  |  |  |  | CC | 20 (8.7) |  |
|  | rs393795  c.653+4065C>A | 0.37 | Intron | In LD with SNPs affecting splicing^g^ | GG | 146 (63.2) | 0.305 |
|  |  |  |  |  | GT | 72 (31.2) |  |
|  |  |  |  |  | TT | 13 (5.6) |  |
| *SLC22A1* | rs628031  p.Met216Val | 0.31 | Coding region | Nonsynonymous may influence splicing**^b^** | GG | 98 (42.4) | 0.587 |
|  |  |  |  |  | GA | 102 (44.2) |  |
|  |  |  |  |  | AA | 31 (13.4) |  |
| *SLC7A5* | rs1060253  c.*438C>G | 0.30 | 3’UTR | May influence miRNA binding**^b^** | CC | 128 (55.4) | 0.031 |
|  |  |  |  |  | CG | 79 (34.2) |  |
|  |  |  |  |  | GG | 24 (10.4) |  |
|  | rs1060257  c.*1282G>A | 0.37 | 3’UTR | May influence miRNA binding**^b^** | GG | 131 (56.7) | 0.026 |
|  |  |  |  |  | GA | 77 (33.3) |  |
|  |  |  |  |  | AA | 23 (10.0) |  |
| *DRD2* | rs1801028  p.Ser311Cys | 0.03 | Coding region | Nonsynonymous  Functional impact on activity^e^ | CC | 221 (95.7) | 0.737 |
|  |  |  |  |  | GC | 10 (4.3) |  |
|  |  |  |  |  | GG | 0 (0.0) |  |
|  | rs1799732  c.-486_-485insC | 0.24 | 5’near gene | Functional impact on expression^e^ | CC | 190 (82.3) | 0.999 |
|  |  |  |  |  | C- | 39 (16.9) |  |
|  |  |  |  |  | -- | 2 (0.9) |  |
| *DRD3* | rs6280  p.Gly9Ser | 0.49 | Coding region | Nonsynonymous may affect splicing**^b^** | TT | 110 (47.6) | 0.111 |
|  |  |  |  |  | TC | 91 (39.4) |  |
|  |  |  |  |  | CC | 30 (13.0) |  |
| *SLC18A2* | rs14240  c.*294T>A | 0.45 | 3’UTR | May influence splicing and miRNA binding | TT | 57 (24.7) | 0.159 |
|  |  |  |  |  | TC | 126 (54.5) |  |
|  |  |  |  |  | CC | 48 (210.8) |  |
| *SV2C* | rs1423099  c.-58C>T | 0.44 | 5’UTR | May influence splicing | CC | 21 (9.1) | 0.490 |
|  |  |  |  |  | CT | 90 (39.0) |  |
|  |  |  |  |  | TT | 120 (51.9) |  |

^a^Genotype missing for one patient.

^b^Evaluated by SNP function prediction.

^c^According to Devos et al., 2014

^d^According to [Hao et al., 2014](#_ENREF_12)

^e^According to [He et al., 2016](#_ENREF_13)

^f^According to dbSNP ([Sherry et al., 2001](#_ENREF_36))

^g^According to [Kaplan et al., 2014](#_ENREF_18)

^h^Calculated only for women due to X chromosome location.

**Supplementary table 2: Excessive daytime sleepiness and sleep attacks, visual hallucinations, and nausea and vomiting**

| **Gene**  **SNP** | **Genotype** | **EDS and sleep attacks** | | | **Visual hallucinations** | | | **Nausea and vomiting** | | |
| --- | --- | --- | --- | --- | --- | --- | --- | --- | --- | --- |
|  |  | **OR** | **95% CI** | **p-value** | **OR** | **95% CI** | **p-value** | **OR** | **95% CI** | **p-value** |
| ***COMT***  **rs4680** | GG | Ref. |  |  | Ref. |  |  | Ref. |  |  |
|  | GA | 1.08 | 0.55-2.11 | 0.835 | 1.20 | 0.55-2.60 | 0.653 | 0.87 | 0.45-1.69 | 0.69 |
|  | AA | 1.66 | 0.79-3.46 | 0.179 | 2.04 | 0.90-4.64 | 0.088 | 0.46 | 0.21-1.03 | 0.059 |
|  | GA + AA | 1.27 | 0.68-2.37 | 0.455 | 1.48 | 0.72-3.04 | 0.283 | 0.70 | 0.38-1.31 | 0.266 |
| ***COMT***  **rs165815** | CC | / |  |  | / |  |  | / |  |  |
|  | CT | / |  |  | / |  |  | / |  |  |
|  | TT | Ref. |  |  | Ref. |  |  | Ref. |  |  |
|  | CC+CT* | 0.65 | 0.36-1.16 | 0.647 | **0.36** | **0.18-0.74** | **0.006** | 0.97 | 0.54-1.75 | 0.917 |
| ***DDC***  **rs921451** | TT | Ref. |  |  | Ref. |  |  | Ref. |  |  |
|  | CT | 1.58 | 0.87-2.88 | 0.132 | 1.19 | 0.62-2.29 | 0.608 | 0.85 | 0.46-1.58 | 0.611 |
|  | CC | 1.00 | 0.46-2.18 | 0.996 | 0.86 | 0.36-2.06 | 0.741 | 0.72 | 0.32-1.61 | 0.424 |
|  | CT+CC | 1.37 | 0.79-2.38 | 0.259 | 1.08 | 0.59-1.97 | 0.809 | 0.81 | 0.46-1.42 | 0.459 |
| ***DDC***  **rs3837091** | AGAGAGAG | Ref. |  |  | Ref. |  |  | Ref. |  |  |
|  | AGAG- | 1.23 | 0.68-2.20 | 0.497 | 0.93 | 0.48-1.78 | 0.828 | 0.80 | 0.44-1.48 | 0.484 |
|  | -- | 0.94 | 0.39-2.25 | 0.882 | 1.21 | 0.49-3.04 | 0.680 | 0.68 | 0.27-1.72 | 0.410 |
|  | AGAG- + -- | 1.15 | 0.67-1.97 | 0.623 | 1.00 | 0.55-1.82 | 0.997 | 0.77 | 0.44-1.35 | 0.363 |
| ***MAOB***  **rs1799836** | AA (male) | Ref. |  |  | Ref. |  |  | Ref. |  |  |
|  | GG (male) | 1.07 | 0.75-1.52 | 0.722 | 0.56 | 0.25-1.28 | 0.172 | 1.09 | 0.47-2.54 | 0.843 |
|  | AA (female) | Ref. |  |  | Ref. |  |  | Ref. |  |  |
|  | AG (female) | 0.62 | 0.24-1.63 | 0.333 | 0.73 | 0.24-2.20 | 0.578 | 0.76 | 0.30-1.91 | 0.558 |
|  | GG (female) | 0.58 | 0.17-2.08 | 0.406 | 0.80 | 0.20-3.23 | 0.754 | 0.46 | 0.14-1.56 | 0.214 |
|  | AG + GG (female) | 0.61 | 0.24-1.53 | 0.290 | 0.75 | 0.27-2.12 | 0.587 | 0.67 | 0.27-1.61 | 0.367 |
| ***SLC6A3***  **rs6347** | AA | Ref. |  |  | Ref. |  |  | Ref. |  |  |
|  | AG | 1.08 | 0.61-1.91 | 0.805 | 0.98 | 0.52-1.87 | 0.960 | 1.19 | 0.66-2.16 | 0.559 |
|  | GG | 0.88 | 0.33-2.32 | 0.793 | 1.46 | 0.54-3.94 | 0.450 | 0.91 | 0.33-2.52 | 0.857 |
|  | AG + GG | 1.03 | 0.60-1.78 | 0.906 | 1.07 | 0.59-1.95 | 0.821 | 1.13 | 0.65-1.99 | 0.663 |
| ***SLC6A3***  **rs1042098** | TT | Ref. |  |  | Ref. |  |  | Ref. |  |  |
|  | TC | 1.03 | 0.58-1.83 | 0.926 | 1.15 | 0.61-2.18 | 0.672 | 1.21 | 0.67-2.18 | 0.530 |
|  | CC | 1.60 | 0.61-4.16 | 0.337 | 1.83 | 0.67-5.02 | 0.243 | 0.81 | 0.27-2.38 | 0.696 |
|  | TC + CC | 1.12 | 0.65-1.93 | 0.682 | 1.26 | 0.69-2.30 | 0.448 | 1.13 | 0.64-1.98 | 0.680 |
| ***SLC6A3***  **rs393795** | GG | Ref. |  |  | Ref. |  |  | Ref. |  |  |
|  | GT | 0.93 | 0.52-1.69 | 0.819 | 0.58 | 0.29-1.17 | 0.127 | 1.08 | 0.59-1.98 | 0.810 |
|  | TT | 0.53 | 0.14-2.00 | 0.346 | 1.17 | 0.34-4.00 | 0.806 | 0.69 | 0.18-2.62 | 0.585 |
|  | GT + TT | 0.86 | 0.49-1.52 | 0.606 | 0.66 | 0.35-1.25 | 0.200 | 1.01 | 0.57-1.81 | 0.969 |
| ***SLC22A1***  **rs628031** | GG | Ref. |  |  | Ref. |  |  | Ref. |  |  |
|  | GA | 0.72 | 0.40-1.30 | 0.273 | 1.12 | 0.58-2.16 | 0.731 | 0.94 | 0.52-1.73 | 0.853 |
|  | AA | 1.19 | 0.52-2.71 | 0.677 | 1.73 | 0.71-4.23 | 0.231 | 1.13 | 0.47-2.71 | 0.779 |
|  | GA + AA | 0.82 | 0.47-1.41 | 0.462 | 1.25 | 0.68-2.30 | 0.480 | 0.99 | 0.56-1.74 | 0.960 |
| ***DRD2***  **rs1801028** | CC | Ref. |  |  | Ref. |  |  | Ref. |  |  |
|  | GC | / |  |  | / |  |  | / |  |  |
|  | GG | / |  |  | / |  |  | / |  |  |
|  | GC + GG | 1.25 | 0.34-4.55 | 0.739 | 0.75 | 0.16-3.64 | 0.721 | 0.99 | 0.25-3.90 | 0.976 |
| ***DRD2***  **rs1799732** | CC | Ref. |  |  | Ref. |  |  | Ref. |  |  |
|  | C- | / |  |  | / |  |  | / |  |  |
|  | -- | / |  |  | / |  |  | / |  |  |
|  | C- + -- | 0.73 | 0.35-1.51 | 0.392 | 1.14 | 0.53-2.45 | 0.738 | 1.23 | 0.60-2.52 | 0.569 |
| ***DRD3***  **rs6280** | TT | Ref. |  |  | Ref. |  |  | Ref. |  |  |
|  | TC | 1.08 | 0.60-1.93 | 0.800 | 1.59 | 0.81-3.12 | 0.174 | 1.07 | 0.58-1.96 | 0.828 |
|  | CC | 0.95 | 0.40-2.23 | 0.901 | **3.40** | **1.43-8.12** | **0.006** | 1.20 | 0.51-2.85 | 0.675 |
|  | TC + CC | 1.05 | 0.61-1.80 | 0.875 | **1.96** | **1.05-3.65** | **0.033** | 1.10 | 0.63-1.94 | 0.736 |
| ***SLC18A2***  **rs14240** | TT | Ref. |  |  | Ref. |  |  | Ref. |  |  |
|  | CT | 1.03 | 0.53-1.98 | 0.935 | 1.05 | 0.52-2.13 | 0.901 | 1.16 | 0.58-2.32 | 0.670 |
|  | CC | 0.93 | 0.41-2.08 | 0.850 | 0.56 | 0.21-1.46 | 0.237 | 1.17 | 0.50-2.70 | 0.722 |
|  | CT+CC | 1.00 | 0.53-1.87 | 0.997 | 0.99 | 0.45-1.78 | 0.757 | 1.16 | 0.60-2.25 | 0.655 |
| ***SV2C***  **rs1423099** | CC | 2.12 | 0.83-5.40 | 0.116 | 0.70 | 0.22-2.34 | 0.698 | 0.89 | 0.32-2.47 | 0.817 |
|  | CT | 0.92 | 0.51-1.64 | 0.767 | 1.02 | 0.54-1.91 | 0.955 | 0.95 | 0.52-1.72 | 0.865 |
|  | TT | Ref. |  |  | Ref. |  |  | Ref. |  |  |
|  | CC+CT* | 1.09 | 0.63-1.86 | 0.766 | 0.95 | 0.52-1.74 | 0.876 | 0.94 | 0.53-1.65 | 0.822 |

*Recessive model was used.

**Supplementary table 3: Orthostatic hypotension, peripheral edema, and impulse control disorders**

| **Gene**  **SNP** | **Genotype** | **Orthostatic hypotension** | | | **Peripheral edema** | | | **Impulse control disorders** | | |
| --- | --- | --- | --- | --- | --- | --- | --- | --- | --- | --- |
|  |  | **OR** | **95% CI** | **p-value** | **OR** | **95% CI** | **p-value** | **OR** | **95% CI** | **p-value** |
| ***COMT***  **rs4680** | GG | Ref. |  |  | Ref. |  |  | Ref. |  |  |
|  | GA | 1.57 | 0.80-3.08 | 0.191 | 1.98 | 0.86-4.54 | 0.109 | 1.99 | 0.69-5.74 | 0.202 |
|  | AA | 1.79 | 0.85-3.77 | 0.124 | 0.84 | 0.30-2.34 | 0.740 | 2.37 | 0.77-7.28 | 0.132 |
|  | GA + AA | 1.65 | 0.88-3.10 | 0.120 | 1.51 | 0.68-3.36 | 0.313 | 2.13 | 0.78-5.81 | 0.140 |
| ***COMT***  **rs165815** | CC | / |  |  | / |  |  | / |  |  |
|  | CT | / |  |  | / |  |  | / |  |  |
|  | TT | Ref. |  |  | Ref. |  |  | Ref. |  |  |
|  | CC+CT* | 0.76 | 0.43-1.35 | 0.352 | 0.96 | 0.48-1.92 | 0.915 | 1.34 | 0.62-2.87 | 0.456 |
| ***DDC***  **rs921451** | TT | Ref. |  |  | Ref. |  |  | Ref. |  |  |
|  | CT | **2.23** | **1.23-4.06** | **0.009** | 1.17 | 0.55-2.50 | 0.691 | 1.76 | 0.79-3.92 | 0.169 |
|  | CC | 1.17 | 0.54-2.52 | 0.695 | 2.10 | 0.89-4.95 | 0.090 | 0.56 | 0.15-2.11 | 0.395 |
|  | CT+CC | **1.82** | **1.05-3.16** | **0.033** | 1.44 | 0.73-2.84 | 0.291 | 1.33 | 0.62-2.88 | 0.463 |
| ***DDC***  **rs3837091** | AGAGAGAG | Ref. |  |  | Ref. |  |  | Ref. |  |  |
|  | AGAG- | **1.79** | **1.00-3.19** | **0.048** | 1.15 | 0.55-2.40 | 0.714 | 1.48 | 0.67-3.25 | 0.334 |
|  | -- | 0.96 | 0.40-2.31 | 0.926 | 2.39 | 0.95-6.04 | 0.065 | 0.85 | 0.23-3.16 | 0.806 |
|  | AGAG- + -- | 1.54 | 0.90-2.62 | 0.117 | 1.43 | 0.74-2.76 | 0.292 | 1.31 | 0.62-2.76 | 0.485 |
| ***MAOB***  **rs1799836** | AA (male) | Ref. |  |  | Ref. |  |  | Ref. |  |  |
|  | GG (male) | 1.10 | 0.54-2.23 | 0.789 | 0.57 | 0.24-1.37 | 0.210 | 0.67 | 0.25-1.79 | 0.425 |
|  | AA (female) | Ref. |  |  | Ref. |  |  | Ref. |  |  |
|  | AG (female) | 1.36 | 0.52-3.60 | 0.533 | 2.03 | 0.52-8.10 | 0.315 | 0.71 | 0.15-3.42 | 0.669 |
|  | GG (female) | 0.56 | 0.15-2.19 | 0.407 | 0.98 | 0.15-6.51 | 0.984 | 1.56 | 0.28-8.72 | 0.611 |
|  | AG + GG (female) | 1.10 | 0.43-2.80 | 0.839 | 1.72 | 0.45-6.65 | 0.429 | 0.93 | 0.22-3.87 | 0.916 |
| ***SLC6A3***  **rs6347** | AA | Ref. |  |  | Ref. |  |  | Ref. |  |  |
|  | AG | 1.24 | 0.70-2.18 | 0.456 | 1.01 | 0.50-2.03 | 0.978 | 0.68 | 0.30-1.55 | 0.360 |
|  | GG | 1.02 | 0.40-2.63 | 0.962 | 0.94 | 0.29-3.03 | 0.914 | 0.84 | 0.23-3.12 | 0.794 |
|  | AG + GG | 1.19 | 0.70-2.04 | 0.515 | 1.00 | 0.52-1.92 | 0.988 | 0.71 | 0.33-1.52 | 0.381 |
| ***SLC6A3***  **rs1042098** | TT | Ref. |  |  | Ref. |  |  | Ref. |  |  |
|  | TC | 1.35 | 0.76-2.38 | 0.306 | 1.20 | 0.60-2.40 | 0.611 | 0.93 | 0.42-2.05 | 0.864 |
|  | CC | 2.44 | 0.94-6.37 | 0.067 | 1.15 | 0.35-3.77 | 0.820 | 0.65 | 0.14-3.04 | 0.582 |
|  | TC + CC | 1.51 | 0.88-2.58 | 0.133 | 1.19 | 0.62-2.29 | 0.607 | 0.88 | 0.41-1.86 | 0.735 |
| ***SLC6A3***  **rs393795** | GG | Ref. |  |  | Ref. |  |  | Ref. |  |  |
|  | GT | 1.39 | 0.78-2.48 | 0.260 | 0.57 | 0.26-1.24 | 0.154 | 1.61 | 0.72-3.62 | 0.247 |
|  | TT | 1.15 | 0.36-3.71 | 0.812 | 0.64 | 0.14-3.05 | 0.577 | 3.58 | 0.99-12.98 | 0.052 |
|  | GT + TT | 1.35 | 0.78-2.34 | 0.279 | 0.58 | 0.28-1.20 | 0.142 | 1.87 | 0.88-3.97 | 0.103 |
| ***SLC22A1***  **rs628031** | GG | Ref. |  |  | Ref. |  |  | Ref. |  |  |
|  | GA | 1.03 | 0.57-1.84 | 0.929 | 0.88 | 0.41-1.90 | 0.752 | 1.40 | 0.59-3.32 | 0.445 |
|  | AA | **2.46** | **1.07-5.66** | **0.034** | **3.92** | **1.60-9.63** | **0.003** | **3.20** | **1.13-9.06** | **0.028** |
|  | GA + AA | 1.26 | 0.73-2.17 | 0.399 | 1.38 | 0.70-2.72 | 0.353 | 1.76 | 0.79-3.91 | 0.165 |
| ***DRD2***  **rs1801028** | CC | Ref. |  |  | Ref. |  |  | Ref. |  |  |
|  | GC | / |  |  | / |  |  | / |  |  |
|  | GG | / |  |  | / |  |  | / |  |  |
|  | GC + GG | 0.69 | 0.18-2.78 | 0.604 | 1.06 | 0.22-5.17 | 0.943 | 0.68 | 0.08-5.54 | 0.716 |
| ***DRD2***  **rs1799732** | CC | Ref. |  |  | Ref. |  |  | Ref. |  |  |
|  | C- | / |  |  | / |  |  | / |  |  |
|  | -- | / |  |  | / |  |  | / |  |  |
|  | C- + -- | 0.63 | 0.30-1.31 | 0.215 | 0.85 | 0.35-2.06 | 0.712 | 0.27 | 0.06-1.19 | 0.083 |
| ***DRD3***  **rs6280** | TT | Ref. |  |  | Ref. |  |  | Ref. |  |  |
|  | TC | 0.60 | 0.33-1.08 | 0.090 | 0.96 | 0.47-1.96 | 0.916 | 0.77 | 0.35-1.69 | 0.512 |
|  | CC | 1.42 | 0.63-3.20 | 0.395 | 1.05 | 0.38-2.89 | 0.928 | 0.36 | 0.08-1.65 | 0.189 |
|  | TC + CC | 0.76 | 0.44-1.29 | 0.305 | 0.98 | 0.51-1.90 | 0.960 | 0.66 | 0.31-1.40 | 0.282 |
| ***SLC18A2***  **rs14240** | TT | Ref. |  |  | Ref. |  |  | Ref. |  |  |
|  | CT | 1.52 | 0.79-2.93 | 0.209 | 1.47 | 0.64-3.37 | 0.363 | 0.66 | 0.29-1.51 | 0.325 |
|  | CC | 0.82 | 0.36-1.89 | 0.647 | 1.07 | 0.38-3.02 | 0.903 | 0.38 | 0.11-1.28 | 0.119 |
|  | CT+CC | 1.30 | 0.69-2.43 | 0.421 | 1.35 | 0.61-3.02 | 0.461 | 0.58 | 0.26-1.29 | 0.179 |
| ***SV2C***  **rs1423099** | CC | 1.54 | 0.60-3.96 | 0.372 | 1.58 | 0.56-4.51 | 0.390 | 1.25 | 0.33-4.79 | 0.745 |
|  | CT | 1.57 | 0.89-2.76 | 0.119 | 0.73 | 0.35-1.51 | 0.393 | 1.50 | 0.68-3.29 | 0.312 |
|  | TT | Ref. |  |  | Ref. |  |  | Ref. |  |  |
|  | CC+CT* | 1.56 | 0.91-2.67 | 0.103 | 0.87 | 0.45-1.68 | 0.679 | 1.45 | 0.68-3.08 | 0.331 |

*Recessive model was used.

**Supplementary table 4: Motor fluctuations and dyskinesia**

| **Gene**  **SNP** | **Genotype** | **Motor fluctuations** | | | **Dyskinesia** | | |
| --- | --- | --- | --- | --- | --- | --- | --- |
|  |  | **OR** | **95% CI** | **p-value** | **OR** | **95% CI** | **p-value** |
| ***COMT***  **rs4680** | GG | Ref. |  |  | Ref. |  |  |
|  | GA | 1.01 | 0.54-1.90 | 0.965 | 0.63 | 0.33-1.20 | 0.160 |
|  | AA | 1.78 | 0.88-3.68 | 0.109 | 1.21 | 0.60-2.46 | 0.590 |
|  | GA + AA | 1.25 | 0.70-2.24 | 0.458 | 0.81 | 0.45-1.46 | 0.484 |
| ***COMT***  **rs165815** | CC | / |  |  | / |  |  |
|  | CT | / |  |  | / |  |  |
|  | TT | Ref. |  |  | Ref. |  |  |
|  | CC+CT* | 0.82 | 0.48-1.41 | 0.472 | 0.79 | 0.46-1.37 | 0.407 |
| ***DDC***  **rs921451** | TT | Ref. |  |  | Ref. |  |  |
|  | CT | 1.29 | 0.73-2.29 | 0.386 | 1.29 | 0.72-2.29 | 0.392 |
|  | CC | 0.96 | 0.47-1.98 | 0.913 | 1.08 | 0.52-2.24 | 0.838 |
|  | CT+CC | 1.17 | 0.70-1.98 | 0.550 | 1.22 | 0.72-2.06 | 0.466 |
| ***DDC***  **rs3837091** | AGAGAGAG | Ref. |  |  | Ref. |  |  |
|  | AGAG- | 1.48 | 0.84-2.62 | 0.176 | **1.83** | **1.04-3.24** | **0.037** |
|  | -- | 0.73 | 0.32-1.66 | 0.448 | 0.73 | 0.31-1.75 | 0.480 |
|  | AGAG- + -- | 1.23 | 0.73-2.07 | 0.434 | 1.46 | 0.87-2.46 | 0.156 |
| ***MAOB***  **rs1799836** | AA (male) | Ref. |  |  | Ref. |  |  |
|  | GG (male) | 0.54 | 0.27-1.09 | 0.085 | 0.67 | 0.33-1.36 | 0.269 |
|  | AA (female) | Ref. |  |  | Ref. |  |  |
|  | AG (female) | 0.73 | 0.29-1.84 | 0.511 | 0.65 | 0.26-1.64 | 0.363 |
|  | GG (female) | 0.64 | 0.20-2.04 | 0.445 | 0.68 | 0.21-2.18 | 0.515 |
|  | AG + GG (female) | 0.71 | 0.29-1.69 | 0.435 | 0.66 | 0.28-1.58 | 0.349 |
| ***SLC6A3***  **rs6347** | AA | Ref. |  |  | Ref. |  |  |
|  | AG | 1.20 | 0.69-2.08 | 0.525 | 0.94 | 0.54-1.64 | 0.835 |
|  | GG | 1.14 | 0.46-2.84 | 0.775 | 0.86 | 0.34-2.16 | 0.747 |
|  | AG + GG | 1.19 | 0.71-1.99 | 0.521 | 0.93 | 0.55-1.56 | 0.771 |
| ***SLC6A3***  **rs1042098** | TT | Ref. |  |  | Ref. |  |  |
|  | TC | 1.35 | 0.78-2.35 | 0.285 | 1.16 | 0.67-2.00 | 0.607 |
|  | CC | 1.22 | 0.47-3.16 | 0.678 | 0.56 | 0.20-1.54 | 0.259 |
|  | TC + CC | 1.33 | 0.79-2.23 | 0.287 | 1.02 | 0.60-1.71 | 0.954 |
| ***SLC6A3***  **rs393795** | GG | Ref. |  |  | Ref. |  |  |
|  | GT | 0.74 | 0.42-1.30 | 0.293 | 0.89 | 0.50-1.58 | 0.689 |
|  | TT | 0.67 | 0.21-2.09 | 0.489 | 1.07 | 0.34-3.33 | 0.910 |
|  | GT + TT | 0.73 | 0.43-1.24 | 0.245 | 0.92 | 0.53-1.57 | 0.749 |
| ***SLC22A1***  **rs628031** | GG | Ref. |  |  | Ref. |  |  |
|  | GA | **0.46** | **0.26-0.81** | **0.007** | **0.43** | **0.24-0.75** | **0.003** |
|  | AA | 1.62 | 0.67-3.88 | 0.282 | 0.70 | 0.31-1.57 | 0.387 |
|  | GA + AA | 0.61 | 0.36-1.04 | 0.070 | **0.48** | **0.28-0.82** | **0.007** |
| ***DRD2***  **rs1801028** | CC | Ref. |  |  | Ref. |  |  |
|  | GC | / |  |  | / |  |  |
|  | GG | / |  |  | / |  |  |
|  | GC + GG | 0.87 | 0.25-3.10 | 0.833 | 0.54 | 0.14-2.13 | 0.378 |
| ***DRD2***  **rs1799732** | CC | Ref. |  |  | Ref. |  |  |
|  | C- | / |  |  | / |  |  |
|  | -- | / |  |  | / |  |  |
|  | C- + -- | 0.71 | 0.36-1.41 | 0.330 | 0.54 | 0.26-1.10 | 0.090 |
| ***DRD3***  **rs6280** | TT | Ref. |  |  | Ref. |  |  |
|  | TC | 1.23 | 0.70-2.15 | 0.468 | 0.98 | 0.56-1.72 | 0.955 |
|  | CC | 1.10 | 0.49-2.47 | 0.814 | 0.60 | 0.26-1.40 | 0.237 |
|  | TC + CC | 1.20 | 0.71-2.01 | 0.497 | 0.87 | 0.52-1.47 | 0.613 |
| ***SLC18A2***  **rs14240** | TT | Ref. |  |  | Ref. |  |  |
|  | CT | 0.92 | 0.49-1.72 | 0.785 | 0.89 | 0.48-1.67 | 0.713 |
|  | CC | 0.72 | 0.33-1.55 | 0.401 | 0.67 | 0.31-1.46 | 0.310 |
|  | CT+CC | 0.86 | 0.47-1.56 | 0.614 | 0.82 | 0.45-1.50 | 0.523 |
| ***SV2C***  **rs1423099** | CC | 0.68 | 0.27-1.73 | 0.417 | 1.02 | 0.40-2.59 | 0.976 |
|  | CT | 1.18 | 0.68-2.05 | 0.549 | 1.13 | 0.65-1.96 | 0.66 |
|  | TT | Ref. |  |  | Ref. |  |  |
|  | CC+CT* | 1.06 | 0.63-1.79 | 0.813 | 1.11 | 0.66-1.87 | 0.697 |

*Recessive model was used.

**Supplementary table 5: Haplotype analysis for the *COMT* gene**

| ***COMT*** | **EDS and sleep attacks** | | **Visual hallucinations** | | **Nausea and vomiting** | | **Orthostatic hypotension** | | **Peripheral oedema** | | **Impulse control disorders** | |
| --- | --- | --- | --- | --- | --- | --- | --- | --- | --- | --- | --- | --- |
| **Haplotype (%)** | **OR**  **(95%CI)** | **p-value** | **OR**  **(95%CI)** | **p-value** | **OR**  **(95%CI)** | **p-value** | **OR**  **(95%CI)** | **p-value** | **OR**  **(95%CI)** | **p-value** | **OR**  **(95%CI)** | **p-value** |
| AT (0.45) | Ref. |  | Ref. |  | Ref. |  | Ref. |  | Ref. |  | Ref. |  |
| GT (0.36) | 0.78  (0.50-1.21) | 0.262 | 0.77  (0.47-1.23) | 0.270 | 1.43  (0.89-2.30) | 0.136 | 0.71  (0.46-1.11) | 0.129 | 1.30  (0.70-2.40) | 0.402 | 0.61  (0.29-1.30) | 0.197 |
| GC (0.14) | 0.56  (0.28-1.11) | 0.096 | **0.34**  **(0.16-0.72)** | **0.005** | 1.41  (0.77-2.60) | 0.268 | 0.76  (0.42-1.36) | 0.351 | 0.85  (0.33-2.16) | 0.730 | 0.84  80.35-2.05) | 0.707 |
| AC (0.06) | 0.47  (0.14-1.64) | 0.237 | 0.42  (0.11-1.65) | 0.217 | 0.87  (0.25-3.04) | 0.828 | 0.71  (0.28-1.80) | 0.466 | 1.75  (0.58-5.25) | 0.319 | 0.97  (0.24-3.99) | 0.971 |
|  | **Motor fluctuations** | | **Dyskinesia** | |  |  |  |  |  |  |  |  |
| **Haplotype (%)** | **OR**  **(95%CI)** | **p-value** | **OR**  **(95%CI)** | **p-value** |  |  |  |  |  |  |  |  |
| AT (0.45) | Ref. |  | Ref. |  |  |  |  |  |  |  |  |  |
| GT (0.36) | 0.78  (0.51-1.20) | 0.261 | 1.06  (0.69-1.61) | 0.791 |  |  |  |  |  |  |  |  |
| GC (0.14) | 0.70  (0.39-1.23) | 0.209 | 0.70  (0.40-1.23) | 0.218 |  |  |  |  |  |  |  |  |
| AC (0.06) | 1.13  (0.43-2.91) | 0.808 | 1.48  (0.58-3.78) | 0.417 |  |  |  |  |  |  |  |  |

**Supplementary table 6: Haplotype analysis for the *DDC* gene**

| ***DDC*** | **EDS and sleep attacks** | | **Visual hallucinations** | | **Nausea and vomiting** | | **Orthostatic hypotension** | | **Peripheral oedema** | | **Impulse control disorders** | |
| --- | --- | --- | --- | --- | --- | --- | --- | --- | --- | --- | --- | --- |
| **Haplotype (%)** | **OR** | **p-value** | **OR** | **p-value** | **OR** | **p-value** | **OR** | **p-value** | **OR** | **p-value** | **OR** | **p-value** |
| TAGAG (0.62) | Ref. |  | Ref. |  | Ref. |  | Ref. |  | Ref. |  | Ref. |  |
| C- (0.29) | 1.08  (0.72-1.63) | 0.713 | 0.99  (0.63-1.56) | 0.980 | 0.78  (0.51-1.21) | 0.266 | 1.19  (0.78-1.79) | 0.417 | 1.49  (0.94-2.37) | 0.089 | 1.07  (0.59-1.95) | 0.813 |
| CAGAG (0.09) | 1.07  (0.55-2.08) | 0.840 | 0.91  (0.41-2.03) | 0.822 | 1.08  (0.55-2.11) | 0.828 | 1.32  (1.70-2.49) | 0.390 | 1.21  (0.56-2.61) | 0.627 | 0.54  (0.14-1.98) | 0.349 |
|  | **Motor fluctuations** | | **Dyskinesia** | |  |  |  |  |  |  |  |  |
| **Haplotype (%)** | **OR** | **p-value** | **OR** | **p-value** |  |  |  |  |  |  |  |  |
| TAGAG (0.62) | Ref. |  | Ref. |  |  |  |  |  |  |  |  |  |
| C- (0.29) | 1.05  (0.72-1.54) | 0.800 | 1.12  (0.76-1.66) | 0.568 |  |  |  |  |  |  |  |  |
| CAGAG (0.09) | 0.95  (0.50-1.77) | 0.863 | 0.93  (0.50-1.76) | 0.832 |  |  |  |  |  |  |  |  |

**Supplementary table 7: Haplotype analysis for the *SLC6A3* gene**

| ***SLC6A3*** | **EDS and sleep attacks** | | **Visual hallucinations** | | **Nausea and vomiting** | | **Orthostatic hypotension** | | **Peripheral oedema** | | **Impulse control disorders** | |
| --- | --- | --- | --- | --- | --- | --- | --- | --- | --- | --- | --- | --- |
| **Haplotype (%)** | **OR** | **p-value** | **OR** | **p-value** | **OR** | **p-value** | **OR** | **p-value** | **OR** | **p-value** | **OR** | **p-value** |
| ATG (0.49) | Ref. |  | Ref. |  | Ref. |  | Ref. |  | Ref. |  | Ref. |  |
| GCG (0.18) | 0.94  (0.54-1.64) | 0.830 | 1.21  (0.68-2.14) | 0.520 | 1.25  (0.72-2.17) | 0.435 | 1.27  (0.73-2.21) | 0.393 | 0.86  (0.42-1.77) | 0.690 | 1.05  (0.40-2.75) | 0.927 |
| ATT (0.15) | 0.67  (0.34-1.32) | 0.242 | 0.78  (0.38-1.61) | 0.507 | 0.98  (0.51-1.89) | 0.948 | 1.31  (0.72-2.40) | 0.383 | 0.66  (0.26-1.71) | 0.395 | **2.08**  **(1.00-4.35)** | **0.051** |
| ACG (0.07) | 1.91  (0.80-4.57) | 0.145 | 0.87  (0.32-2.34) | 0.781 | 0.61  (0.23-1.61) | 0.315 | **2.48**  **(1.01-6.07)** | **0.047** | 1.95  (0.74-5.14) | 0.175 | 0.51  (0.09-2.89) | 0.447 |
| GTG (0.06) | 0.61  (0.18-2.00) | 0.412 | 0.66  (0.20-2.22) | 0.503 | 0.43  (0.12-1.58) | 0.202 | 0.80  (0.27-2.37) | 0.690 | 1.50  (0.50-4.50) | 0.472 | 0.97  (0.25-3.84) | 0.966 |
|  | **Motor fluctuations** | | **Dyskinesia** | |  |  |  |  |  |  |  |  |
| **Haplotype (%)** | **OR** | **p-value** | **OR** | **p-value** |  |  |  |  |  |  |  |  |
| ATG (0.49) | Ref. |  | Ref. |  |  |  |  |  |  |  |  |  |
| GCG (0.18) | 1.32  (0.77-2.27) | 0.308 | 0.88  (0.52-1.50) | 0.650 |  |  |  |  |  |  |  |  |
| ATT (0.15) | 0.91  (0.51-1.62) | 0.750 | 0.85  (0.47-1.52) | 0.580 |  |  |  |  |  |  |  |  |
| ACG (0.07) | 1.24  (0.52-2.92) | 0.628 | 0.62  (0.26-1.48) | 0.280 |  |  |  |  |  |  |  |  |
| GTG (0.06) | 1.08  (0.42-2.73) | 0.876 | 0.65  (0.24-1.75) | 0.398 |  |  |  |  |  |  |  |  |

**Supplementary table 8: Haplotype analysis for the *DRD2* gene**

| ***DRD2*** | **EDS and sleep attacks** | | **Visual hallucinations** | | **Nausea and vomiting** | | **Orthostatic hypotension** | | **Peripheral oedema** | | **Impulse control disorders** | |
| --- | --- | --- | --- | --- | --- | --- | --- | --- | --- | --- | --- | --- |
| **Haplotype (%)** | **OR**  **(95%CI)** | **p-value** | **OR**  **(95%CI)** | **p-value** | **OR**  **(95%CI)** | **p-value** | **OR**  **(95%CI)** | **p-value** | **OR**  **(95%CI)** | **p-value** | **OR**  **(95%CI)** | **p-value** |
| CC | Ref. |  | Ref. |  | Ref. |  | Ref. |  | Ref. |  | Ref. |  |
| C- | 0.70  (0.34-1.45) | 0.341 | 1.20  (0.60-2.39) | 0.614 | 1.12  (0.56-2.22) | 0.757 | 0.61  (0.29-1.26) | 0.180 | 1.13  (0.56-2.29) | 0.729 | 0.28  (0.06-1.21) | 0.089 |
| ***DRD2*** | **Motor fluctuations** | | **Dyskinesia** | |  |  |  |  |  |  |  |  |
| **Haplotype (%)** | **OR**  **(95%CI)** | **p-value** | **OR**  **(95%CI)** | **p-value** |  |  |  |  |  |  |  |  |
| CC | Ref. |  | Ref. |  |  |  |  |  |  |  |  |  |
| C- | 0.75  (0.40-1.40) | 0.366 | 0.60  (0.31-1.14) | 0.119 |  |  |  |  |  |  |  |  |

**
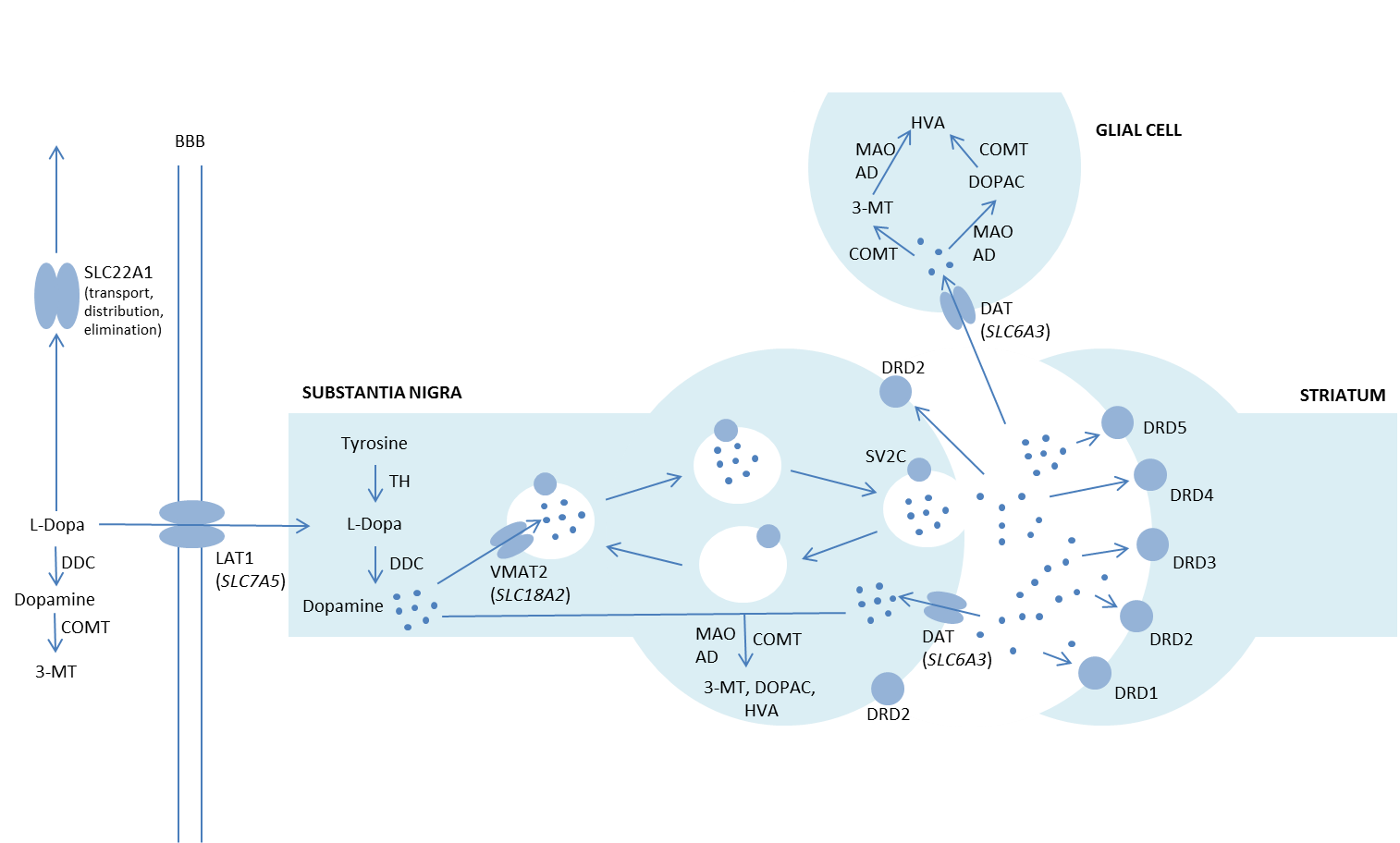
**

**Supplementary figure 1: Schematic figure of the dopaminergic system**

© 2018 REDENŠEK Sara, TROŠT Maja, DOLŽAN Vita. Adapted from Redenšek et al., 2018; originally published under CC BY 3.0 license. Available from: 10.5772/intechopen.75051

**
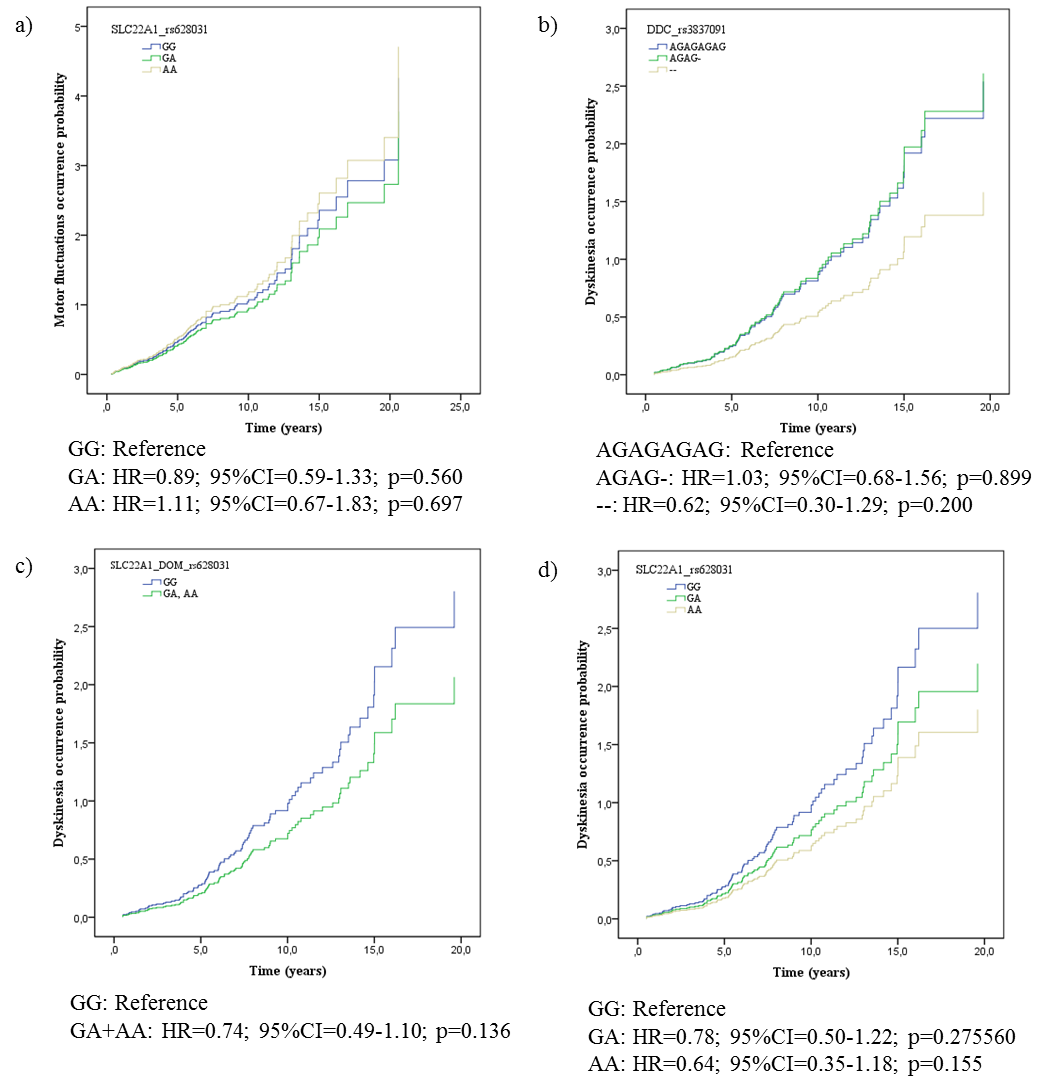
**

**Supplementary figure 2: Results of survival analysis for significant associations after univariate logistic regression analysis**
